# Use of rheumatology-specific patient navigators to understand and reduce barriers to medication adherence: Analysis of qualitative findings

**DOI:** 10.1371/journal.pone.0200886

**Published:** 2018-07-19

**Authors:** Alyssa Wohlfahrt, Anarosa Campos, Maura D. Iversen, Joshua J. Gagne, Elena Massarotti, Daniel H. Solomon, Candace H. Feldman

**Affiliations:** 1 Department of Medicine, Division of Rheumatology, Immunology and Allergy, Brigham and Women’s Hospital, Boston, MA, United States of America; 2 Department of Physical Therapy, Northeastern University, Movement and Rehabilitation Sciences, Boston, MA, United States of America; 3 Department of Medicine, Division of Pharmacoepidemiology and Pharmacoeconomics, Brigham and Women’s Hospital, Boston, MA, United States of America; Sint Maartenskliniek, NETHERLANDS

## Abstract

**Objective:**

Adherence to medications among patients with rheumatic diseases is often suboptimal. Patient navigators, individuals trained in care coordination, motivational interviewing and basic rheumatology and pharmacology, have not been employed to explore and address this issue. We piloted a single-site, single arm intervention to determine the feasibility and acceptability of using rheumatology-specific navigators to understand and reduce barriers to adherence to oral disease modifying anti-rheumatic drugs (DMARDs). We analyzed our qualitative findings from navigator-patient interactions as well as patient satisfaction with the intervention.

**Methods:**

We recruited patients ≥18 years with a systemic rheumatic disease who initiated an oral DMARD within the prior 6 months. Navigators conducted baseline needs assessments and 2–4 week follow-up calls to understand and address issues related to medication adherence. We analyzed patient-navigator encounters qualitatively using content analysis to identify key themes related to barriers to adherence and navigator actions performed in response to the barriers described. We also categorized intentional and unintentional nonadherent behavior and assessed satisfaction with the navigator experience (range 0–5, 5 = most satisfied).

**Results:**

107 rheumatology patients were followed for up to 6 months. Mean patient age was 55 years (+17) and 93% were female; 36% described one or more episode of intentional or unintentional nonadherence. The three most common themes identified as barriers to adherence were fear of adverse events (raised by 54%), concerns about medication effectiveness (43%), and challenges with medication acquisition (32%). 86% of participants described at least one adherence-related barrier. Frequent navigator actions included facilitation of patient-doctor communication (38%), medication and diagnosis education (27%), and development of individualized strategies to improve adherence (16%). Patients were satisfied with the navigator experience (mean 4.4 + 0.9).

**Conclusion:**

Navigators uncovered and addressed a number of medication adherence-related concerns and patients were satisfied with the services provided.

## Introduction

In chronic autoimmune diseases, like rheumatoid arthritis (RA) and systemic lupus erythematosus (SLE), medication adherence is essential for disease control and prevention of n**e**gative outcomes. Inadequate medication adherence can lead to increased RA disease activity, poorer function, risk of co-morbid conditions and early mortality.[1–5] Among patients with SLE and RA, inconsistent and poor medication use can lead to a rise in health care costs[6, 7] and acute care utilization.[8] Despite this, current rates of adherence for rheumatology patients are suboptimal; adherence to oral DMARDs and steroid medication regimens range from 58% to 71%, with only one-fifth of patients showing greater than 80% adherence.

Prior studies have characterized nonadherence as intentional, or a purposeful action, and unintentional, or a passive behavior (e.g. forgetting to take medication), the latter of which is less strongly associated with beliefs.[9–12] Studies have demonstrated that among initiators of chronic disease medications, intentional nonadherers had lower perceptions of the necessity of their medication and greater concerns about taking their medications.[10, 13] Medication adherence is a complex behavior affected by patient, provider and health system factors, and interactions between these units.[14] Previous interventions targeting both intentional and unintentional nonadherence among patients with rheumatic diseases have had limited success possibly due in part, to a lack of understanding of the nuances of adherence behavior over time, and each patient’s unique reasons for nonadherence.[15]

Our goal was to assess the feasibility of and patient experience with a patient navigator as an intervention to understand and address disease-modifying antirheumatic drug (DMARD) nonadherence. Our patient navigators were non-healthcare professionals trained in motivational interviewing, advocacy, basic medical knowledge and care coordination who aimed to better understand and respond to barriers to medication adherence among patients with rheumatic diseases.[16] We hypothesized that an individual other than the prescribing physician might be able to understand and track adherence behavior, and respond to issues that both directly and indirectly affect medication use. Patient navigators, have been used in other chronic disease populations to help patients overcome barriers to their care and improve health outcomes.[16] Patient navigators have been particularly beneficial to racial/ethnic minorities, low income patients, and non-English speaking patients.[17] We developed and piloted a single-arm patient navigator intervention to provide longitudinal, nonjudgmental contact with rheumatology patients to better understand personal barriers to DMARD adherence and to intervene using strategies designed for each patient’s needs. We collected detailed qualitative data regarding the patient and navigator experience with the intervention presented here, and quantitative pre- and post- intervention surveys, which we described separately.[18]

## Materials and methods

We conducted a single-arm, single site pilot quality improvement intervention to assess the feasibility and acceptability of a patient navigator to understand and address adherence to oral DMARDs among patients with rheumatic diseases.

### Patient identification and consent

Participants were recruited from the Brigham and Women’s Hospital (BWH) rheumatology clinic. We included English and Spanish-speaking patients age ≥18 years with a diagnosis of any systemic rheumatic disease and documentation of oral DMARD initiation within the prior 6 months without any additional exclusion criteria. Patients meeting these criteria were identified using electronic medical record review and the primary rheumatologist was approached to introduce the study in person or by letter to the patient if the rheumatologist felt it to be appropriate. In addition, all practicing rheumatologists were informed of the nature of the intervention simultaneously at a division-wide conference and were able to directly refer patients who met criteria but may not have been identified via electronic medical record review if they felt the patients might benefit from having a navigator involved in their care. The Partners Healthcare Institutional Review Board approved the study (IRB Protocol #2013P002334). Verbal informed consent was obtained from all participants and all data were stored and analyses were performed using de-identified data. Patients are referred to in this manuscript by their study ID number to maintain anonymity.

### Patient navigator selection and training

Two college graduates (AC, AW) without prior medical training but with experience conducting rheumatology-related research studies involving patients at the BWH rheumatology clinic, served as the patient navigators. One navigator (AC) was a native Spanish speaker. Two board certified rheumatologists (CHF, DHS), a behavioral scientist (MDI) and a licensed pharmacist (JG), designed and led a project-specific week-long training program for both navigators and MDI, along with CHF and DS were available at weekly meetings to discuss issues and themes. The training had four main components: basic DMARD pharmacology, fundamentals of systemic rheumatic diseases, motivational interviewing and resource awareness. DMARD pharmacology training included information about dosing, adverse events and monitoring, as well as prior authorizations, automated refill systems, mail order pharmacy procedures. Rheumatic disease-related training was provided regarding disease manifestations and warning signs of disease activity and medication side effects. The navigators also received a two-day motivational interviewing training led by a behavioral scientist (MDI). Motivational interviewing has been used successfully as part of disease management programs when treatment plans rely on behavior.[19] Motivational interview training included role-playing patient scenarios of common adherence issues and debriefing the experience. The goal was to train navigators in the interview technique and alert them to areas of concern specific to rheumatology patients.

**Fig 1:**
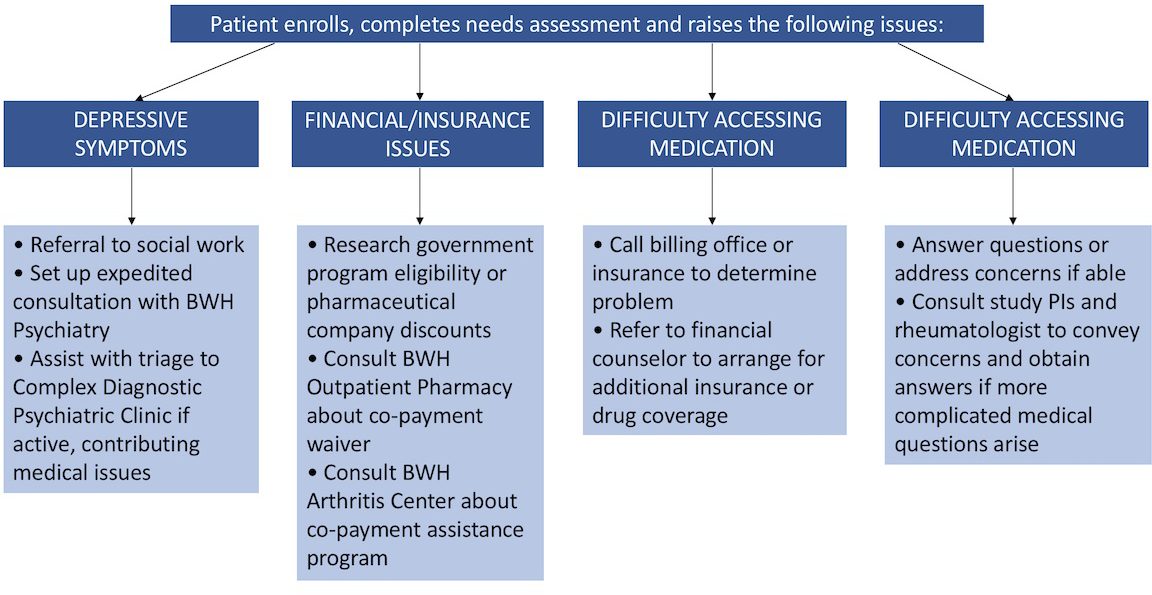
Flow chart of rheumatology-specific patient navigator action plan.

The navigators also met with key personnel including the practice administrator and three clinical rheumatologists, to better understand and facilitate care coordination. At these meetings, the navigators learned about clinic practice flow and the rheumatologists’ perspectives about medication-related issues. To facilitate referrals, the navigators also met with the hospital’s financial counselor, the department’s social worker, and psychiatry leadership. The navigators shadowed rheumatology nurse practitioners to observe medication-related conversations. From these meetings, a flow chart was developed to guide the actions of the navigators based on the needs of patients and the services available (**Fig 1**).

### Patient navigator pilot intervention design

The study intervention period was six months. At the baseline visit or phone conversation, the patient navigators conducted a semi-structured interview following a prespecified question guide with each interview lasting from 15 to 25 minutes. Interviews assessed the patient’s knowledge of his/her disease and medications, adherence, and adherence-related barriers (S1 File). Specifically, the navigators asked patients which oral DMARDs they were prescribed, how they were taking their medications, and if they had any medication-related concerns. For patients who described nonadherence, the navigators asked follow-up questions to determine the etiologies. The navigators also addressed the patients’ readiness to improve their adherence. Questions were grounded in motivational interviewing principles and therefore conversations were unique to each patient.

In situations where patients raised specific barriers to adherence, navigators worked with the patients to develop a series of potential strategies to overcome these obstacles. The navigator obtained permission from the patient before any actions were taken, such as contacting the patient’s rheumatologist. Navigators followed the flow chart shown in **Fig 1**to address common problems presented by patients. These strategies were developed prior to the intervention and were further enhanced based on the patients’ needs. To maintain intervention fidelity, the navigators met weekly in person with the study PIs to review their patient interactions, their call notes, any barriers they encountered, and the actions performed in accordance with the pre-specified flow chart. Due to the individually-tailored nature of the intervention, at times unique needs were uncovered and additional patient-specific actions were performed.

Following the baseline meeting, navigators contacted patients via telephone, in person after clinic visits, or via e-mail, depending on patient preferences. Interactions with patients lasted between 10 to 25 minutes depending on need. For those patients struggling with adherence, navigators reached out approximately every 2 weeks; patients without specific needs were contacted monthly. During each follow-up interaction, navigators asked a standardized series of open-ended questions to guide the conversation (S2 File). These questions included: “1) What has it been like taking your medication for your rheumatic disease? 2) Have you missed any doses of your medication for any reason or had any trouble remembering to take it? If so, when and why did this occur? 3) Have you experienced any side effects or symptoms recently that have been out of the ordinary? and 4) Do you have any questions or concerns at this time?” Due to the nature of the navigator-patient interactions, it was not feasible to transcribe verbatim each conversation. Part of the goal of the intervention was for the navigators to develop relationships with the participants and to use motivational interviewing principles to guide the interactions. Transcribing these conversations would have impeded the spontaneous, dynamic and organic nature of the communication between the navigator and the patient and having a separate researcher present to collect data during these interactions would have altered the intervention. Therefore, navigators provided detailed free-form text documentation of all patient interactions (calls, emails, or in-person meetings) both during and immediately following each interaction, key points were revisited with each participant, and notes were discussed and reviewed at weekly study team meetings. Baseline and 6-month quantitative surveys to assess adherence, disease activity, mental health and beliefs about medications were also collected for a subset of participants and findings have been described previously.[18]

Six months following the baseline assessment, patients were asked to complete a patient satisfaction survey (S3 File). Patients were asked to rate their satisfaction with the intervention (scale of 1–5, 1 = unsatisfied, 5 = satisfied) and to identify the best aspects of the program as well as the ways in which the intervention could be improved.

### Data analyses

Content analysis was used to analyze 84 typed pages of patient interaction notes. This process included identification of categories and refinement of themes arising from the text related to the study aims, establishing rules for coding text, categorizing text and refinement of the coding process, and theme identification using an iterative process.[20, 21] Specifically, five authors (AW, AC, MDI, DHS, and CHF) first read through all notes independently and identified themes in two overarching categories: medication-related issues raised by patients and navigator actions performed. Within each category, dominant themes and subthemes emerged. Themes and subthemes were discussed at an in-person meeting using a normative group process to develop group consensus. Each team member then independently re-coded the data using these themes. The team met in person again to adjudicate differences in text coding; final assignment of a theme required consensus of two or more study members. Notes for an individual patient frequently included more than one theme; however, once a theme was assigned to a patient it was not recounted, even if it emerged in a later call. To quantify our findings to inform future interventions, a total count reflecting the number of times a theme was identified was performed for the study cohort.

We also characterized patients’ descriptions of their nonadherence behavior as intentional or unintentional. [9–11] We defined intentional nonadherence as the conscious choice not to take doses of a medication and grouped unintentional nonadherence as patient-related (e.g. forgetfulness), treatment-related (e.g. difficulty with refills), and patient-provider related (e.g. miscommunication about doses).[10] Four authors (AW, AC, DHS and CHF) also agreed upon four illustrative vignettes to highlight the nuanced nature of issues encountered and the work of the navigators. We analyzed data from the patient satisfaction survey using the mean + SD for ordinal answers and counted responses for each of the descriptive answers.

## Results

Among 384 patients who were invited to participate, we enrolled 107 patients; enrollment status required at least one conversation with the navigator. Of these patients, 98 (92%) engaged with the navigator following this initial conversation. Reasons for lack of ongoing engagement included switching to a non-oral DMARD, expression of lack of need for assistance, or inability to re-contact the patient. Participants were predominately female (93.5%) with a mean age of 55 + 17 years (**Table 1**). The majority were white (70%), and privately insured (53%). Most were diagnosed with RA (81%), and 56 had recently initiated methotrexate.

**Table 1:** Baseline characteristics of patients enrolled in the patient navigator intervention.

Characteristics	N = 107
**Female–N (%)**	100 (93.5)
**Mean age, years (±SD)**	54.7 (±16.7)
**Race–N (%)**	
White	75 (70.1)
Asian	2 (1.9)
Black/African American	6 (5.6)
Not reported^a^	24 (22.4)
**Ethnicity–N (%)**	
Non-Hispanic	83 (77.6)
Hispanic	19 (17.8)
Not reported	5 (4.7)
**Primary Language–N (%)**	
English	92 (86.0)
Spanish	13 (12.2)
Bilingual	2 (1.9)
**Insurance Status–N (%)**	
Medicaid	14 (13.1)
Medicare	35 (32.7)
Private	57 (53.3)
Other	1 (0.9)
**Education–N (%)**	
Graduated College or graduate school	50 (46.7)
Some College	24 (22.4)
High School/GED	18 (16.8)
Some High School	3 (2.8)
8^th^ Grade or Less	3 (2.8)
Decline/Unknown/Not reported	9 (8.4)
**Rheumatic Disease–N (%)**	
Rheumatoid Arthritis	87 (81.3)
Lupus	7 (6.5)
Mixed Connective Tissue Disease	4 (3.7)
Other	9 (8.4)
**DMARD Use^b^ –N (%)**	
Methotrexate	56 (52.3)
Sulfasalazine	11 (10.3)
Tofacitinib	7 (6.5)
Hydroxychloroquine	40 (37.4)

^**a**^Most patients of Hispanic ethnicity did not report race

^b^Categories are not mutually exclusive; patients received more than one DMARD simultaneously

### Intentional and unintentional nonadherence

Thirty-nine (36.4%) patients described at least one episode of either intentional or unintentional nonadherence, with one patient reporting both. [9, 10] Sixteen patients described intentional nonadherence. For 11 of the patients, the primary reason for intentional nonadherence was due to side effects. Three patients expressed a fear of side effects, one patient felt appropriate education about the medication was not provided, one patient felt the medication was ineffective, and one patient felt overwhelmed and opted to discontinue all her medications.

Twenty-four patients described unintentional nonadherence to one or more of the medications prescribed by their rheumatologist. The primary patient-related factor was forgetting to take medications (18 patients). Three patients stated that they forgot in the setting of social situations; one patient forgot on the days she worked. Two patients forgot their morning doses when they overslept and two patients stated that any deviation to their usual routine would cause them to forget. One patient took additional doses of her medications because she was not sure if she took them earlier that day. Four patients described treatment-related issues notably that they were unable to take their medications as prescribed because of insurance-related problems obtaining refills. Three patients highlighted patient-provider related issues; they were confused by the directions given and were uncertain whether they were taking their medications correctly.

### Medication-related themes

Seven key themes of medication-related issues (**Table 2**) emerged based on content analysis of the detailed navigator notes. Eighty-six percent of participants identified at least one concern, with the most prevalent (54%) being medication-related adverse events. Eighteen patients described nausea, stomach upset or abdominal pain, eight felt that their medications increased their fatigue or caused “brain fog” and six reported hair loss or thinning. Other patients reported infection or fear of infection (6 patients), depression/agitation (4), headaches (4), blurry vision (2), mouth sores (2), rash (1), nail discoloration (1) and sexual side effects (1). Three patients expressed a general fear of taking medications that have the potential to cause serious side effects, two were concerned about eye toxicity, and one person expressed a fear of developing cancer from the medication prescribed.

**Table 2:** Themes of medication-related issues elicited by the navigator.

Themes of Medication-related Issues Raised with the Navigator	Percent of patients (N = 107) expressing issue ≥1 time	Subcategories	Illustrative examples
Medication-related adverse events	54.2	1a. Experience of medication-related adverse events (gastrointestinal symptoms and hair loss)	1a. Patient 41 reported having stomach pain and a flu-like illness, which she felt were related to leflunomide use. She stopped this medication on her own and then let her physician know.
			1a. Patient 78 said she felt nauseous for about three days after she took her methotrexate and got diarrhea later in the day after she took her methotrexate.
		1b. Fear of experiencing adverse events or association of possibly unrelated symptoms with medications	1b. Patient 81 described blurry vision, headaches and abnormally yellow urine. Her rheumatologist checked lab tests and told her it was unlikely to be related to her DMARD. She was told to restart her DMARD. However, she began taking the medication every other day because she felt that it was probably the cause of her symptoms.
Difficulty with medications	31.8	1a. Difficulty obtaining or refilling medications	1a. Patient 1 reported that a family member threw out her medication by accident and was unable to refill it because her insurance would not cover it until the subsequent month. She said she also could not afford the $106 out of pocket fee.
			1a. Patient 25 reported forgetting to order refills and found it inconvenient and burdensome that she could only fill one month at a time.
		1b. Difficulty taking medications	1b. Patient 48 reported she forgot her medications because of memory issues, which she attributed to her lupus.
Concerns with medication effectiveness	43.0	1a. Delays from initiation to clinical improvement	1a. Patient 50 continued to experience pain and had been to the emergency room because of it. She felt better when she took steroids but she did not feel that her methotrexate was starting to work and wanted to be switched to a different medication.
		1b. Medication was working but symptoms returned, particularly when tapering off concomitant prednisone	1b. Patient 95 was doing well but felt like some of her symptoms were coming back as she tapered her prednisone dosage.
			1b. Patient 94 felt a little stiff but better when on 5mg of prednisone. He was concerned about having more pain as he tapered his prednisone dose down further.
		1c. Switches between medications and general concerns about effectiveness	1c. Patient 41 was switched from methotrexate to leflunomide but thought she felt worse since the switch with increased exhaustion, shortness of breath and tender lymph nodes. She expressed a desire to go back on the methotrexate instead.
**Lack of knowledge**	21.5	1a. Inadequate information about medication or disease	1a. Patient 39 expressed confusion as to whether her knee and back pain was related to her RA or to something else. She was not sure whether her DMARD was working because she had been prescribed other medications (duloxetine and gabapentin) as well as an epidural injection to treat her pain.
			1a. Patient 89 continued to feel a lot of pain in her hands and she was not sure if the DMARD was working or whether her pain was related to osteoarthritis and not to her rheumatoid arthritis.
Need for emotional and social support and/or mental health services	17.7	1a. Expressions of stress, sadness, frustration with disease or medications	1a. Patient 1 was distraught that her mother was fired from her job because she had repeatedly left work to either care for her or take her to appointments related to her RA.
		1b. Description of depressive symptoms and desire for psychiatric services	1b. Patient 63 described feeling discouraged about her medications not working and stated that she felt depressed. She said she felt moody and fragile and as though her emotions were not anchored. She expressed interest in a referral for mental health services as well as for a support group.
**Financial/insurance related issues**	15.9		Patient 57 reported having to switch to Medicare in order to get disability and her medication, tofacitinib would not be covered. She expressed concern about her options and whether she needed to obtain supplemental coverage. She was also concerned that her rheumatologist would not take her new insurance.
Interruptions in medication use	13.1	1a. Physician-instructed (e.g. in the case of surgery, pregnancy or infection)	1a. Patient 57 reported being diagnosed with a lung infection or rheumatoid lung and was told her hold her DMARD for several months.
		1b. Patient-initiated because of lack of effectiveness	1b. Patient 41 felt that she had more pain, stiffness, and fatigue and she found it increasingly difficult to go up hills. She felt her DMARD was not working and had not taken it for the past couple of days to see whether there was a difference when she did not take it.
		1c. Discontinuation because patient feels well	1c. Patient 26 reported no longer feeling any symptoms from her RA and therefore self-discontinued her DMARD.

Thirty-two percent of patients described challenges obtaining or physically taking their medication. These included difficulties filling prescribed medications or obtaining refills, questions regarding when to take medications, need for reminders about when to take medications, or challenges obtaining a prior authorization. Forty-three percent of patients had concerns about medication effectiveness, including the amount of time they would need to wait before observing a difference in symptoms. Twenty-one percent of patients expressed lack of knowledge about their medication or diagnosis. Patients raised issues about the long-term effects of DMARD use (3 patients), about the timing of administration (e.g. taking the medications with meals) (2), dietary changes that might impact their disease or their medication use (3), and the use of vitamins (4). Fourteen percent of patients did not report any medication-related issues.

### Navigator action-related themes

We identified seven key themes of navigator actions in response to issues raised by participants (**Table 3**). The most frequent navigator action was to facilitate communication, with the patient’s permission, with the treating rheumatologist (38%). Most patients felt they were being bothersome if they reached out to their rheumatologist directly. Patient navigators also provided education about the patients’ rheumatic diseases and their medications, specifically potential side effects and need for regular monitoring (e.g. ophthalmology appointments in the case of hydroxychloroquine).

**Table 3:** Themes of actions performed by the navigators in response to issues raised by patients.

Themes of Actions Performed by the Navigators	Percent of patients for whom actions were performed (N = 107)	Example of issue raised by patient	Navigator action performed in response
**Facilitation of patient-doctor communication**	38.3	Patient 1 had an ultrasound that she was very concerned about and wanted to speak with her physician about the results but had not heard back.	Navigator communicated this to the patient’s rheumatologist who then contacted the patient.
		Patient 48 was concerned about memory issues and wanted to see a neurologist. However, she had missed prior appointments and was told she could no longer reschedule.	Navigator reached out to the neurology department without success and then contacted the patient’s rheumatologist who was able to facilitate an appointment.
		Patient 27 was prescribed a medication for pain but felt that it made her extremely tired	Navigator spoke with the patient’s rheumatologist who sent in a lower dose of the pain medication for her to try instead.
**Medication and diagnosis education**	27.1	Patient 70 was concerned about retinal toxicity with hydroxychloroquine.	Navigator explained that this was rare, particularly in the first years of use, but that she needed to continue with annual eye exams to ensure that she was screened.
		Patient 28 felt like she was never educated on long-term effects or side effects of sulfasalazine and therefore she did not want to take this medication.	Navigator provided information sheets in Spanish about sulfasalazine to the patient.
**Development of individualized strategies to improve adherence**	15.9	Patient 2 did not want to keep her pills out because she did not want others to know about her illness and carried her medications in her purse. She tried alarm reminders but felt they did not work and that they were annoying. She was interested in a different strategy.	Navigator sent text messages to remind the patient to take her medications.
**Assistance with financial/insurance issues**	15.0	Patient 76 received bills for thousands of dollars for laboratory tests and appointments with her rheumatologist. She began receiving calls from a collection agency to pay her bills.	Navigator called the patient’s insurance company, the hospital billing department and the collection agency and discovered a billing error and resolved the issue.
**Care coordination**	15.0	Patient 71 had been followed by rheumatology, orthopedics and podiatry and recently had foot surgery. She had been unable to obtain the boot that she needed in order to walk. She had missed multiple appointments in part because of this. She had not been able to successfully communicate with any of her providers, with the medical supply store or with her insurance.	Navigator contacted insurance company, medical supply company, podiatrist and orthopedist, got all of the necessary referrals, prior authorizations and prescriptions sent appropriately and was able to get the patient the necessary boot and shoe inserts that she needed.
**Social and emotional support**	12.1	Patient 57 described having multiple tests to understand the etiology of her shortness of breath and all were normal. She felt very frustrated with her care and did not want to talk to physicians for a while.	Navigator provided regular calls to the patient and encouragement to have her continue to see and speak with her doctors. The patient did not want the navigator to communicate with her doctors on her behalf and therefore the navigator did not do this, but regularly called the patient to listen to her concerns.
**Social work and psychiatry referrals**	8.4	Patient 63 described feeling discouraged and depressed about her illness and her medications.	Navigator communicated this to the patient’s primary rheumatologist to help facilitate a referral to a therapist. Navigator also investigated local support groups and provided the patient with this information.

Based on the unique barriers or concerns of each patient, the navigators worked with the patient to develop individualized strategies to improve adherence (16%). Examples of strategies discussed with patients included the use of pillboxes (5 patients), reminder text messages (1 patient), automatic refill setup (3 patients), placement of a reminder magnet on the refrigerator (1 patient) and development of a medication schedule to minimize forgetfulness (4 patients). The navigators also assisted with financial and insurance-related issues to reduce the cost of medication copayments, obtain referrals or facilitate enrollment in safety-net drug insurance plans or emergency coverage (15%). At the end of the 6-month intervention, there were no financial or insurance issues that were raised by the patients related to their medications that were not resolved by the navigator or by the navigator’s referral to the hospital’s financial counselor.

### Patient vignettes

We present four illustrative cases demonstrating interactions between patients and navigators (**Table 4**). Patients 2 and 16 highlight the variety of medication-related issues and individually-tailored strategies used to help patients adhere to their medications. Patient 2 is intentionally nonadherent due to side effects and communication facilitated by the navigator resulted in a new regimen. Patient 16 struggled both with intentional and unintentional nonadherence and the navigator’s ability to communicate in Spanish facilitated the relationship. Patient 31 provides an example related more broadly to a patient’s interactions with the healthcare system. The case of Patient 62 demonstrates a complex situation where the role of the navigator was less straightforward and may have complicated the patient-rheumatologist relationship.

**Table 4:** Four selected patient vignettes with key themes highlighted.

Patient ID	Vignette	Key Themes
2	31 year-old woman with SLE, described difficulty remembering to take her hydroxychloroquine. The navigator sent daily text message reminders to the patient to take her pills, and provided biweekly communication to assess her adherence. Pentoxifylline was also started and the navigator helped the patient integrate this into her daily regimen. The patient developed side effects (nausea and dizziness) and self-discontinued the medication. With the patient’s permission, the navigator promptly informed the patient’s rheumatologist and facilitated a new regimen for the patient.	Difficulty with medications, medication-related adverse events, development of individualized strategies to improve adherence, facilitation of patient-doctor communication
16	24 year-old Spanish-speaking woman with SLE who described difficulty taking 10 different medications. She complained of intermittent stomach upset, which prevented her from adhering to a number of her medications including azathioprine. She also described frequent colds and she would hold her medications when she felt ill. Additionally, she would sleep late when she did not feel well and therefore miss the morning doses of many of her medications. The navigator explained indications to hold her medications and encouraged her to alert her rheumatologist any time she did so. The navigator also communicated these episodes of non-adherence to the rheumatologist with the patient’s permission. After discussions with the primary rheumatologist, the navigator also suggested that she take certain medications later in the day with dinner to minimize stomach upset and to ensure she was adherent even when she slept late. The patient also described difficult getting a primary care appointment and obtaining a necessary cardiology referral, in part due to a language barrier, and the Spanish-speaking navigator facilitated both.	Difficulty with medications, medication-related adverse events, interruptions in medication adherence, lack of knowledge, facilitation of patient-doctor communication, care coordination, development of individualized strategies to improve adherence
31	79 year-old woman with inflammatory arthritis prescribed methotrexate, reported to the navigator that she received bills from the hospital for her arthritis care that she was unable to pay. She was hesitant to return for further care due to fear of continued bills. The navigator contacted the hospital’s billing department and the patient’s insurance company and found that the bills were an error. Ultimately, the amount charged to the patient was reduced to an affordable level. The navigator’s understanding of the health care system allowed for continued care that was not financially prohibitive.	Financial/insurance related issues and assistance with these issues
62	82 year-old female with multiple comorbidities prescribed mycophenolate mofetil to treat systemic sclerosis, described difficulty taking her complex medication regimen. The patient also had difficulty obtaining refills from her pharmacy, was experiencing side effects from her medications, and had concerns about taking some of her medications. As a result, she had decided to modify her dosing and stop taking some of her medications, without consulting her physicians. The navigator reached out to the patient weekly to better understand the side effects she was experiencing and the medication changes she was making. With the patient’s permission, the navigator was then able to relay information to her rheumatologist, who monitored the patient for safety issues, and consulted with her other physicians to develop a treatment plan with which the patient agreed. Her rheumatologist also reached out to the patient more frequently to address her concerns. The navigator also arranged for automated pharmacy refills. During follow-up conversations with the navigator, the patient expressed a number of issues with other medications she was taking including omeprazole, metoprolol, and lisinopril. She asked a number of questions about these medications, their indications, and the need for her to continue to take them. The navigator conveyed these questions and concerns to the patient’s rheumatologist who expressed some frustration feeling that these medications had repeatedly been discussed at length with the patient. Ultimately the rheumatologist asked that the patient be removed from the study as he felt this back and forth information resulted in mixed messages for the patient and made her care more difficult to manage.	Difficulty with medications, medication-related adverse events, interruptions in medication adherence, lack of knowledge, facilitation of patient-doctor communication, development of individualized strategies to improve adherence

### Patient satisfaction survey

Eighty-three patients (78%) completed the patient satisfaction survey at the end of 6 months of enrollment in this study (**Table 5**). On a scale of 1 (unsatisfied) to 5 (satisfied), the mean+ SD score was 4.4 + 0.9. Patients were asked about the best aspects of the program and were able to choose more than one response. The primary benefit highlighted by 39 patients was the ability to communicate with someone about their disease. Patients were also asked how the program could be improved. Fourteen patients felt that more services were needed to help get them through the healthcare system and in general, patients favored an expansion of the navigator’s role.

**Table 5:** Patient satisfaction survey results (N = 83).

Survey question	Response options	Responses (Number)
Identify the best aspects of the program*	a) Help understanding my medications	a) 20
	b) Help understanding and coping with my disease	b) 19
	c) Having a person to talk to about my disease	c) 39
	d) Improved communication with my rheumatologist	d) 15
	e) Reminders to take my medication	e) 3
	f) Help getting and refilling my medications	f) 5
	g) Help with insurance difficulties	g) 6
	h) Help getting through the health care system	h) 16
	i) Other	i) Other responses included: “hope”, “think about every aspect of my disease and how I improved,” “knowing someone cares about me and is interested in my health,” “follow-up by a real person made me cared for” and “being able to help with research.”
Identify ways in which the intervention could be improved*	a) More frequent calls/emails	a) 10
	b) Less frequent calls/emails	b) 10
	c) More help with understanding my medications	c) 6
	d) More help understanding my disease	d) 10
	e) Help with medications for other diseases	e) 8
	f) Navigator accompaniment to my rheumatology appointments	f) 11
	g) More services to help get through the healthcare system	g) 14

*Respondents could choose more than one option

## Discussion

We developed and implemented a patient navigator intervention among patients with systemic rheumatic diseases who recently initiated a DMARD. We demonstrated the feasibility of training non-healthcare professionals to understand and monitor intentional and unintentional DMARD nonadherence and to develop individually tailored strategies to help patients deal with medication and healthcare-related issues. On average, patients expressed satisfaction with the navigator intervention and most provided positive feedback regarding the navigator’s role.

Our study enabled us to observe the way in which DMARD adherence may be affected by potentially modifiable patient, provider, treatment and healthcare system factors.[14] The nuanced barriers to adherence uncovered by the navigators reflected the complexity of rheumatic disease management and the need for individually-tailored adherence interventions. Previous interventions have used one-size-fits-all strategies, such as an educational campaign [15] or a group-based arthritis self-management program,[22] and may not be able to address patient-specific etiologies of nonadherence. The interventions with a trend toward improved adherence used approaches that targeted adherence barriers and behavior at the individual level.[23]

While 36 percent of patients in our study reported episodes of either intentional or unintentional nonadherence to their medications, 86 percent described medication-related issues with the potential to influence their future adherence. The themes of medication-related issues uncovered by the navigators paralleled those in prior qualitative studies.[24–26] Notably, perceptions about medications, experiences with and side effects from medications, information about medications, and need for informational, practical, social and emotional support have been described.[24–26] Interestingly, a prior study showed only weak associations between number of side effects experienced and beliefs about medication necessity and nonadherence behavior, and no association between satisfaction with information received, medication concerns or coping styles.[27] The authors hypothesized that there was no single dominant risk factor that explained nonadherence behavior and therefore, individualized approaches to understand and address barriers may be beneficial.[27] Our findings presented here, albeit qualitative, were similar. The navigators performed a wide range of actions in response to the varied concerns raised by participants. Some services were directly related to adherence (e.g. text message reminders or physician communication about side effects to change regimens). However other actions were indirectly related such as obtaining a post-surgical boot to help a patient walk to her follow-up appointments and pick up her medications.

Similar to prior studies in patients with rheumatic diseases, more patients in our study described unintentional nonadherence than intentional, with the most frequent reason being forgetting to take the medication as prescribed.[28] We suspect that rates of nonadherence may be higher than reported. Some patients may either have been hesitant to completely disclose nonadherent behavior to the navigator, or may not have viewed skipping a few doses of their prescribed medication as significant. A number of patients describe medication-related side effects, although this did not translate into nonadherence in all of the patients. It is possible that the navigators’ frequent interactions with patients, their suggestions of strategies to overcome certain common side effects, and facilitation of communication with rheumatologists to alter doses and regimens when indicated, may have prevented future nonadherence. While it was not possible to directly assess the number of times during which the navigator uncovered issues that were not raised during the rheumatology visit, from the interactions the navigators had, for the majority of the rheumatologists, it was clear significant additional information was obtained.

The navigators experienced an important challenge that resulted in the withdrawal of one patient from the study (Vignette ID 62). The navigators saw their role both as patient advocates and as medication adherence advocates and in one instance, the two roles conflicted. In this case, the patient had a number of concerns about her medications and the navigator tried to address them with the patient and with the rheumatologist. The patient felt she had previously raised these issues with the rheumatologist who felt he had already explained the necessity of each medication and expressed frustration with the navigator’s involvement. The navigator was torn between advocating for the patient to ensure that her concerns were addressed, and understanding the rheumatologist’s perspective that there were no other options to prevent complications from her disease. In this situation, it is possible that the navigator may have added complexity to her care.

In the development of this intervention, we learned that a non-medical professional who received basic training in pharmacology, rheumatology and motivational interviewing could fill the navigator role. Possibly, because patients knew that the navigator was not a medical professional, the role they were asked to play rarely required additional medical knowledge. The availability of the principal investigators who were both physicians, to the navigators was sufficient for the few occasions when more urgent medical assistance was needed. A few patients also expressed that they felt more comfortable sharing concerns with navigators and perceived that communication with their rheumatologist would have been a burden. This seemed to be especially true among patients who felt more comfortable conversing in Spanish with the Spanish-speaking navigator. In these cases, the care coordination the navigator provided seemed particularly valuable. While further studies are needed to examine the cost-effectiveness of a patient navigator, the ability of non-healthcare professionals to serve in this navigator role is important to consider.

This study was limited by our use of only two navigators, which resulted in large caseloads. Our patient population was predominately, white, non-Hispanic, with high school or greater levels of education and with rheumatoid arthritis, which is representative of our clinic population. However, the findings of this pilot study are unlikely to be generalizable across all clinic populations, rheumatic diseases, racial/ethnic groups, or literacy levels. We did find that many of the adherence and healthcare-related barriers the navigators uncovered in this population paralleled those found in prior studies among patients with rheumatic diseases. While the navigators received training in motivational interviewing, it is possible that patients were hesitant to completely reveal nonadherence behavior or the issues they faced. In addition, response bias may have further developed over the course of the intervention due to a desire to please the navigators and demonstrate appreciation for their efforts. The navigators carefully documented all patient interactions during and immediately following their conversations however this may be subject to recall bias. Notably, there may have been underrepresentation of both barriers and actions performed among the most complex patients. There was also loss to follow-up during the intervention which largely resulted from patients deciding they did not need the assistance, and many patients who were contacted with letters by mail inviting them to participate did not respond. This was a pilot study and the primary goal was to understand the feasibility and acceptability of this intervention, and secondarily, to attempt to improve the quality of care provided to our patient population. Therefore, we did not have a control arm, and we allowed patients to be referred directly by their rheumatologist if they felt their patient might benefit from having a navigator involved in their care. There were no resources available in our clinic to help patients with potentially increased needs to navigate the healthcare system or obtain and adhere to their medication. Therefore, we felt that the potential benefit of this intervention to those in need outweighed the selection bias this may have introduced. In addition, we felt that understanding the feasibility and acceptability of this intervention among those with increased needs would pave the way for a future randomized controlled trial that specifically targeted the highest risk patients for whom this intervention would likely be most applicable and cost-effective. The intervention lasted only six months, which may not be sufficient to change long-term behavior or outcomes. The navigators were also limited in their ability to integrate into clinical practice and comprehensively improve patient care, as they did not attend physician visits, did not check electronic medical record notes, and primarily communicated with patients by telephone. In addition, our intervention only enrolled patients who newly started oral DMARDs; we were unable to determine challenges and barriers faced by patients on infusion or subcutaneously administered medications.

Our study showed that patient navigators were able to engage with patients newly initiating oral DMARDs to understand their medication adherence and the medication and healthcare-related obstacles they faced. While more patients described concerns related to their medications than described intentional or unintentional nonadherence, the navigators were able to perform a variety of tasks that both directly and indirectly addressed adherence. This tailored approach may be the most beneficial for the patient and in many cases, may also enhance the patient-provider relationship, facilitate improved communication, and prevent potentially avoidable future medication discontinuation and adverse outcomes.

## Supporting information

S1 File Navigator semi-structured interview guide for baseline and 6-month navigator-patient interaction. (PDF)

S2 File Interview guide for all navigator-patient interactions other than baseline and 6-months. (PDF)

S3 File Patient satisfaction survey. (PDF)

## References

[1] Abu-Shakra M, Urowitz MB, Gladman DD, Gough J. Mortality studies in systemic lupus erythematosus. Results from a single center. I. Causes of death. The Journal of rheumatology. 1995;22(7):1259–64. . 7562755

[2] Wolfe F, Mitchell DM, Sibley JT, Fries JF, Bloch DA, Williams CA, et al The mortality of rheumatoid arthritis. Arthritis and rheumatism. 1994;37(4):481–94. . 8147925 10.1002/art.1780370408

[3] Waimann CA, Marengo MF, de Achaval S, Cox VL, Garcia-Gonzalez A, Reveille JD, et al Electronic monitoring of oral therapies in ethnically diverse and economically disadvantaged patients with rheumatoid arthritis: consequences of low adherence. Arthritis Rheum. 2013;65(6):1421–9. 10.1002/art.37917 ; PubMed Central PMCID: PMC3691007. 23728826 PMC3691007

[4] Cannon GW, Mikuls TR, Hayden CL, Ying J, Curtis JR, Reimold AM, et al Merging Veterans Affairs rheumatoid arthritis registry and pharmacy data to assess methotrexate adherence and disease activity in clinical practice. Arthritis Care Res (Hoboken). 2011;63(12):1680–90. 10.1002/acr.20629 . 21905260 PMC5497696

[5] Innala L, Moller B, Ljung L, Magnusson S, Smedby T, Sodergren A, et al Cardiovascular events in early RA are a result of inflammatory burden and traditional risk factors: a five year prospective study. Arthritis Res Ther. 2011;13(4):R131 10.1186/ar3442 ; PubMed Central PMCID: PMC3239373. 21843325 PMC3239373

[6] Julian LJ, Yelin E, Yazdany J, Panopalis P, Trupin L, Criswell LA, et al Depression, medication adherence, and service utilization in systemic lupus erythematosus. Arthritis Rheum. 2009;61(2):240–6. 10.1002/art.24236 ; PubMed Central PMCID: PMC2875189. 19177526 PMC2875189

[7] de Achaval S, Suarez-Almazor ME. Improving treatment adherence in patients with rheumatologic disease. J Musculoskelet Med. 2010;27(10). ; PubMed Central PMCID: PMC3782860. 24078770 PMC3782860

[8] Feldman CH, Yazdany J, Guan H, Solomon DH, Costenbader KH. Medication Nonadherence Is Associated With Increased Subsequent Acute Care Utilization Among Medicaid Beneficiaries With Systemic Lupus Erythematosus. Arthritis Care Res (Hoboken). 2015;67(12):1712–21. 10.1002/acr.22636 ; PubMed Central PMCID: PMC4684806. 26097166 PMC4684806

[9] Lehane E, McCarthy G. An examination of the intentional and unintentional aspects of medication non-adherence in patients diagnosed with hypertension. Journal of clinical nursing. 2007;16(4):698–706. 10.1111/j.1365-2702.2005.01538.x . 17402951

[10] Lehane E, McCarthy G. Intentional and unintentional medication non-adherence: a comprehensive framework for clinical research and practice? A discussion paper. International journal of nursing studies. 2007;44(8):1468–77. 10.1016/j.ijnurstu.2006.07.010 . 16973166

[11] Lowry KP, Dudley TK, Oddone EZ, Bosworth HB. Intentional and unintentional nonadherence to antihypertensive medication. Ann Pharmacother. 2005;39(7–8):1198–203. 10.1345/aph.1E594 . 15956238

[12] Wroe AL. Intentional and unintentional nonadherence: a study of decision making. J Behav Med. 2002;25(4):355–72. . 12136497 10.1023/a:1015866415552

[13] Clifford S, Barber N, Horne R. Understanding different beliefs held by adherers, unintentional nonadherers, and intentional nonadherers: application of the Necessity-Concerns Framework. J Psychosom Res. 2008;64(1):41–6. 10.1016/j.jpsychores.2007.05.004 . 18157998

[14] Osterberg L, Blaschke T. Adherence to medication. N Engl J Med. 2005;353(5):487–97. 10.1056/NEJMra050100 . 16079372

[15] Galo JS, Mehat P, Rai SK, Avina-Zubieta A, De Vera MA. What are the effects of medication adherence interventions in rheumatic diseases: a systematic review. Ann Rheum Dis. 2016;75(4):667–73. 10.1136/annrheumdis-2014-206593 . 25667208

[16] Kelly E, Ivers N, Zawi R, Barnieh L, Manns B, Lorenzetti DL, et al Patient navigators for people with chronic disease: protocol for a systematic review and meta-analysis. Systematic reviews. 4:28 10.1186/s13643-015-0019-1 . 25874724 PMC4375835

[17] Lasser KE, Kenst KS, Quintiliani LM, Wiener RS, Murillo J, Pbert L, et al Patient navigation to promote smoking cessation among low-income primary care patients: a pilot randomized controlled trial. J Ethn Subst Abuse. 2013;12(4):374–90. 10.1080/15332640.2013.819311 ; PubMed Central PMCID: PMCPMC3827692. 24215228 PMC3827692

[18] Feldman CH, Wohlfahrt A, Campos A, Gagne JJ, Iversen MD, Massarotti E, et al Can Patient Navigators Improve Adherence to Disease-Modifying Antirheumatic Drugs? Quantitative Findings From a Six-Month Single-Arm Pilot Intervention. Arthritis Care Res (Hoboken). 2017 10.1002/acr.23302 . 28575545

[19] Rubak S, Sandbaek A, Lauritzen T, Christensen B. Motivational interviewing: a systematic review and meta-analysis. Br J Gen Pract. 2005;55(513):305–12. . 15826439 PMC1463134

[20] Sandelowski M, Barroso J. Classifying the findings in qualitative studies. Qual Health Res. 2003;13(7):905–23. 10.1177/1049732303253488 . 14502957

[21] Cho JY, Lee E. Reducing Confusion about Grounded Theory and Qualitative Content Analysis: Similarities and Differences. The Qualitative Report. 2014;19(32):1–20.

[22] Conn DL, Pan Y, Easley KA, Comeau DL, Carlone JP, Culler SD, et al The effect of the Arthritis Self-Management Program on outcome in African Americans with rheumatoid arthritis served by a public hospital. Clinical rheumatology. 32(1):49–59. 10.1007/s10067-012-2090-5 . 23053684

[23] Rapoff MA, Belmont J, Lindsley C, Olson N, Morris J, Padur J. Prevention of nonadherence to nonsteroidal anti-inflammatory medications for newly diagnosed patients with juvenile rheumatoid arthritis. Health Psychol. 2002;21(6):620–3. . 12433016 10.1037//0278-6133.21.6.620

[24] Pasma A, van 't Spijker A, Luime JJ, Walter MJ, Busschbach JJ, Hazes JM. Facilitators and barriers to adherence in the initiation phase of Disease-modifying Antirheumatic Drug (DMARD) use in patients with arthritis who recently started their first DMARD treatment. J Rheumatol. 2015;42(3):379–85. 10.3899/jrheum.140693 . 25512473

[25] Brandstetter S, Hertig S, Loss J, Ehrenstein B, Apfelbacher C. 'The lesser of two evils…'—views of persons with rheumatoid arthritis on medication adherence: a qualitative study. Psychol Health. 2016;31(6):675–92. 10.1080/08870446.2016.1139111 . 26832226

[26] Mathijssen EG, Vriezekolk JE, Eijsbouts AM, van den Hoogen FH, van den Bemt BJ. Support needs for medication use and the suitability of eHealth technologies to address these needs: a focus group study of older patients with rheumatoid arthritis. Patient Prefer Adherence. 2018;12:349–58. 10.2147/PPA.S152759 ; PubMed Central PMCID: PMCPMC5846299. 29563778 PMC5846299

[27] van den Bemt BJ, van den Hoogen FH, Benraad B, Hekster YA, van Riel PL, van Lankveld W. Adherence rates and associations with nonadherence in patients with rheumatoid arthritis using disease modifying antirheumatic drugs. J Rheumatol. 2009;36(10):2164–70. 10.3899/jrheum.081204 . 19723906

[28] Daleboudt GM, Broadbent E, McQueen F, Kaptein AA. Intentional and unintentional treatment nonadherence in patients with systemic lupus erythematosus. Arthritis Care Res (Hoboken). 2011;63(3):342–50. 10.1002/acr.20411 . 21120967

