# Supplementary material for: Use of rheumatology-specific patient navigators to understand and reduce barriers to medication adherence: Analysis of qualitative findings
Source: PLoS One. 2018 Jul 19;13(7):e0200886. doi: 10.1371/journal.pone.0200886 (PMC6053216; doi:10.1371/journal.pone.0200886)
Supplement: S1 File — (PDF) [file pone.0200886.s001.pdf]

S1 File

Navigator Semi-Structured Interview Guide for Baseline and 6-month Navigator-Patient Interaction

Record ID:
Date:
Visit/Call number:
Navigator Initials:

What is the name of the DMARD you are taking?

Have you been taking your DMARD?

<u>For patients having difficulty with adherence:</u>
What are the challenges preventing you from taking your medication as prescribed?
Potential prompts:

- *It is difficult for me to remember*
- *I am worried about side effects*
- *I do not think the medication helps me*
- *I am having trouble paying for my medication*
- *I don't have time to take my medication*
- *I have too much going on in my life*
- *I take too many medications and can't keep track of them all*

Please describe the other challenges preventing you from taking your medications as prescribed.
Navigator notes: ____

What are some strategies that you think you can use to help you take your medication as prescribed?
Potential prompts:

- *I can set a reminder (on phone, with placement of a pill box, on calendar)*
- *I can combine taking my medication with something else I do in my daily routine (take when have coffee after breakfast, keep in my car for drive to work)*
- *I can talk to my doctor or a nurse about my concerns*
- *I can organize all my medications so I can remember to take them*
- *I can remind myself what life is like without my medication*
- *I can take other actions to feel healthier (exercise, talk to a therapist)*
- *I can rely on emotional support and/or reminders from my loved ones*

Please describe an additional strategy you would like to use.
Navigator notes: ____

<u>For patients taking their medication as prescribed:</u>
How would you say taking your medication as prescribed is going?

Potential prompts:

- *Excellent*
- *Good*
- *Fair*
- *Poor*
- *Badly*

Please explain:

____

How frequently do you take your DMARD?

- Bi-weekly
- Weekly
- Twice/week
- Daily
- PRN
- Other

What time of day do you take your medication?

- AM
- Mid-day
- PM

Notes regarding timing of medication: ____

How did you decide on that time? ____

What do you use to help you remember to take this medicine?

Potential prompts:

- *Family/friend involvement*
- *Phone/email/calendar reminders*
- *Placement*
- *Pillbox*
- *Taking medication to work*
- *Other*

Please explain: ____

Everyone forgets to take their medications every now and then. When that happens, do you remember why? ____

How many medications are you currently taking? _____________

[If above number is >3]: **Has it been hard for you to figure out the timing of when to take these medications?**

How did you decide on your schedule? Please explain.

We are trying to think of ways to help people who have some difficulty taking their medications regularly and would like your help. What do you think might be the hardest thing for them?

Do you have any ideas of things we could do to help?

Can you think of anything they could do on their own?

<u>Plan</u>

Please state the plan mutually decided upon between the patient and the navigator to improve or continue medication adherence

Indicate whether patient is adherent based on above conversation.

- *Yes*
- *No*
