# Supplementary material for: Use of rheumatology-specific patient navigators to understand and reduce barriers to medication adherence: Analysis of qualitative findings
Source: PLoS One. 2018 Jul 19;13(7):e0200886. doi: 10.1371/journal.pone.0200886 (PMC6053216; doi:10.1371/journal.pone.0200886)
Supplement: S2 File — (PDF) [file pone.0200886.s002.pdf]

S2. Interview Guide for all Navigator-Patient Interactions Other than Baseline and 6-months

Record ID:
Date:
Visit/Call number:
Navigator Initials:

What is the name of the DMARD you are taking?

Have you been taking your DMARD?

What has it been like taking your medication for your rheumatic disease?

Have you missed any doses of your medication for any reason or had any trouble remembering to take it? If so, when and why did this occur?

Have you experienced any side effects or symptoms recently that have been out of the ordinary?

Do you have any questions or concerns at this time?"
