# Supplementary material for: Use of rheumatology-specific patient navigators to understand and reduce barriers to medication adherence: Analysis of qualitative findings
Source: PLoS One. 2018 Jul 19;13(7):e0200886. doi: 10.1371/journal.pone.0200886 (PMC6053216; doi:10.1371/journal.pone.0200886)
Supplement: S3 File — (PDF) [file pone.0200886.s003.pdf]

S3. Patient Satisfaction Survey

Thank you for participating in the Med Assist Program. We hope you will fill out this survey to tell us about your experience working with us and any ways we can improve the program.

1. Please rate your satisfaction with the Med Assist program.
   1(unsatisfied)   2   3   4   5 (very satisfied)

2. What was the best aspect of the program (Can choose more than one)?
   a) Help understanding my medications (when to take them, possible side effects)
   b) Help understanding and coping with my disease (i.e. rheumatoid arthritis, lupus)
   c) Having a person to talk to about my disease
   d) Improved communication with my rheumatologist
   e) Reminders to take my medication
   f) Help getting and refilling my medications
   g) Help with insurance difficulties (including paying for my medications)
   h) Help getting through the health care system (i.e. figuring out who to call when I needed help)
   i) Other: ____

3. How could we improve the Med Assist program?
   a) More frequent calls/emails
   b) Less frequent calls/emails
   c) More help with understanding my medications and how to take them
   d) More help understanding my disease
   e) Help with medications for other diseases
   f) Having the Med Assist navigator accompany me to my rheumatology appointments
   g) More services helping me get through the healthcare system in addition to my rheumatology care
   h) Other: ____

4. Please rate your understanding of your rheumatic disease.
1 (don't understand at all)   2   3   4   5 (understand completely)

5. Please rate your understanding of the medications you take for your rheumatic disease.
1 (don't understand at all)   2   3   4   5 (understand completely)

5. Please rate your understanding of the resources available to you at Brigham and Women's and/or Faulkner Hospital's Arthritis Centers.

1 (don't understand at all) 2 3 4 5 (understand completely)

Thank you for participating in the Med Assist program. Any additional comments/suggestions would be appreciated:

____
____
____
